# Understanding vaccine acceptance and demand—and ways to increase them

**DOI:** 10.1007/s00103-019-03063-0

**Published:** 2019-12-04

**Authors:** Katrine Bach Habersaat, Cath Jackson

**Affiliations:** 1 World Health Organization Regional Office for Europe, Marmorvej 51, 2100 Copenhagen, Denmark; 2 Valid Research Limited, Wetherby, UK

**Keywords:** Tailoring Immunization Programmes (TIP), Immunization, Behavioural insights, Vaccination uptake, Vaccine hesitancy, Länderspezifische Anpassung von Impfprogrammen, Impfung, Verhaltensbezogene Erkenntnisse, Annahme des Impfangebots, Impfmüdigkeit

## Abstract

Vaccination saves millions of lives, and the World Health Organization (WHO) European Region celebrated record high coverage in 2018. Still, national or sub-national coverage is insufficient to stop the spread of vaccine-preventable diseases. Health authorities are increasingly aware of the need to prioritize the “demand” side of vaccination. Achieving high and equitable vaccination uptake in all population groups is not a quick-fix; it requires long-term investment in multifaceted interventions, informed by research with the target groups. The WHO focuses on both individual and context determinants of vaccination behaviours. Individual determinants include risk perceptions, (dis)trust and perceived constraints; insights from psychology help us understand these. Context determinants include social norms, socioeconomic status and education level, and the way health systems are designed, operate and are financed. The WHO recommends using a proven theoretical model to understand vaccination behaviours and has adapted the “COM‑B model” for their Tailoring Immunization Programmes (TIP) approach. This adapted model is described in the article. Informed by insights into the factors affecting vaccination behaviours, interventions and policies can be planned to increase vaccination uptake. Some evidence exists on proven methods to do this. At the individual level, some interventions have been seen to increase vaccination uptake, and experimental studies have assessed how certain messages or actions affect vaccination perceptions. At the context level, there is more documentation for effective strategies, including those that focus on making vaccination the easy, convenient and default behaviour and that focus on the interaction between caregivers and health workers.

## Zusammenfassung

Impfungen retten Millionen von Leben, und die europäische Region der Weltgesundheitsorganisation (WHO) feierte im Jahr 2017 eine Rekordhöhe in Bezug auf die erreichten Impfquoten. Dennoch sind die nationalen und subnationalen Impfquoten weiterhin nicht ausreichend, um die Verbreitung von impfpräventablen Erkrankungen wie Masern zu stoppen. Die Gesundheitsbehörden werden sich zunehmend der Notwendigkeit bewusst, die „Nachfrage“ in Bezug auf Impfungen zu priorisieren. Hohe und gerecht verteilte Impfquoten in allen Bevölkerungsgruppen sind nicht innerhalb kurzer Zeit zu erreichen; sie erfordern langfristige Investitionen in vielfältige Maßnahmen, die auf Studienergebnissen aus den Zielgruppen beruhen. Die WHO folgt der Empfehlung, den Fokus sowohl auf individuelle als auch auf kontextbezogene Determinanten des Impfverhaltens zu setzen. Zu den individuellen Determinanten gehören Risikowahrnehmung, Misstrauen bzw. Vertrauen und wahrgenommene Hürden; Erkenntnisse aus der Psychologie helfen, diese zu verstehen. Kontextdeterminanten schließen kulturelle Faktoren, soziale Normen, den sozioökonomischen Status und das Bildungsniveau sowie die Struktur des Gesundheitssystems ein. Die WHO empfiehlt zum besseren Verständnis des Impfverhaltens die Anwendung eines anerkannten theoretischen Modells und hat das bewährte COM-B-Modell für ihren Tailoring-Immunization-Programmes(TIP)-Ansatz angepasst. Dieses Modell wird im vorliegenden Beitrag beschrieben. Auf der Grundlage von Studien in Zielgruppen können Interventionen konzipiert werden. Es gibt Evidenz für die Verbesserung der Impfraten durch Best-Practice-Modelle und bewährte Methoden. Auf individueller Ebene konnte für einige Maßnahmen eine Steigerung der Inanspruchnahme von Impfungen nachgewiesen werden, während experimentelle Studien gezeigt haben, wie bestimmte Botschaften oder Maßnahmen die Sichtweise auf Impfungen beeinflussen. Auf der Kontextebene gibt es mehr Belege für effektive Strategien zur Steigerung der Impfquoten, einschließlich solcher, die sich darauf konzentrieren, Impfung zum einfachen, bequemen und gewohnten Verhalten zu machen, und die sich auf die persönliche Interaktion zwischen Eltern/Betreuer und Gesundheitspersonal konzentrieren.

**Fig. 1:**
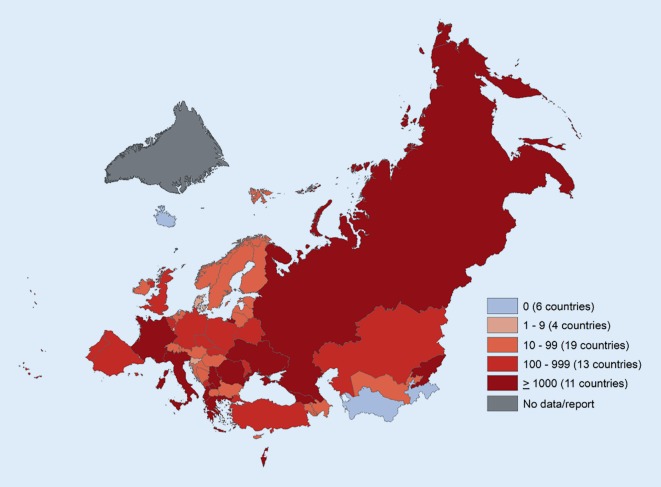
Reported measles cases in the WHO European Region, 2018. Disclaimer: The boundaries and names shown and the designations used on this map do not imply the expression of any opinion whatsoever on the part of the World Health Organization concerning the legal status of any country, territory, city or area or of its authorities, or concerning the delimitation of its frontiers or boundaries. Dotted and dashed lines on maps represent approximate border lines for which there may not yet be full agreement. (© WHO 2019. All rights reserved)

Vaccination is without doubt one of the most outstanding health inventions, saving millions of lives every year, and sparing children and adults from painful disease and absence from education and work [1]. It is also one of the success stories of health systems in the World Health Organization (WHO) European Region, whose 53 Member States in 2018 celebrated record high coverage for the second dose of measles vaccine of 91% and 94% for the third dose of diphtheria–tetanus–pertussis vaccine [2]. Still, some individuals and population groups remain unvaccinated, or are not fully or timely vaccinated. Current measles outbreaks, with cases in almost all of the Member States in the Region (see Fig. 1; [3]), bear witness that vaccination rates at national or sub-national levels continue to be insufficient to ensure community protection and stop the spread of vaccine-preventable diseases.

Whose responsibility is this? Member States and international organizations have jointly set ambitious goals for health and vaccination in the European Region and beyond [4, 5]. These goals depend on people being vaccinated, and so to reach them, health authorities have a critical task in offering vaccination services as well as in ensuring easy and convenient access, supporting and promoting vaccination, and ensuring equitable extension of vaccination services to all [4].

Recognizing this responsibility, health authorities across the region are increasingly aware of the need to give more attention to what has become known as the “demand” (recipient/caregiver) side of vaccination, and many are reaching out to international organizations such as the European Centre for Disease Prevention and Control (ECDC), UNICEF and WHO for support and guidance in this field.

The WHO and our partner organizations have developed tools to support Member States in addressing vaccine acceptance and demand (defined as, ranging from passive acceptance to active demand [6]). We recommend to first identify the most important challenges and barriers to vaccination, applying a broad perspective at the outset; and then to gradually prioritize and focus on the identified core of the problem when identifying ways to increase uptake. This process may take some time, but it ensures that interventions are evidence-informed and tailored to the local context meaning that they are more likely to be effective and represent a cost-effective investment.

Applying a broad perspective at the outset has two overall implications. It is about recognizing that both individual and context determinants influence vaccination behaviours; and it is about using a comprehensive theoretical model that warrants that all potential barriers are considered, leaving no “blind spots” in the analysis.

## Individual and context determinants of vaccination

In the public debate, parental concerns about vaccine safety, myths and misperceptions about vaccination and distrust in health authorities are often highlighted as key reasons for low vaccination uptake and current measles outbreaks. An increasing volume of scientific research has indeed shown how individual risk perception and (dis)trust affect vaccination decisions and behaviours (negatively and positively), and how these factors are guided by often unconscious heuristics, effects and biases—that is, individual mental shortcuts that help us sort impressions and information and make decisions in our information-packed everyday life [7]. As an example, any human being tends to make decisions based on intuition and judge the likelihood of events by the ease with which they come to our mind (availability bias; [7]). This means that possible side effects of a vaccine may feel more likely than the disease it prevents, as they would happen immediately and as a consequence of action, not in a distant future. It is difficult to understand or compare risk of vaccination versus risk of disease. As a result, some people fear the vaccine more than the disease. These insights from psychology are critical to understand individual reasons behind vaccine hesitancy [8] and for designing effective messages and education within information activities with the intended positive outcome. Even more critically, they can help avoid acting in ways which backfire, create more distrust or further confirm misperceptions about vaccination [9].

However, drawing on this often fascinating insight into the human mind and how messages and education may affect it might lead health authorities to apply a cognitive deficit approach [10]—meaning to assume that sufficient knowledge will lead a person to vaccinate—and to assume that immunization uptake can be increased through effective information campaigns alone. This ignores the multiple other determinants of vaccination intentions and behaviours. In fact, a comprehensive literature review concluded that relying on information as the primary means to influence vaccination behaviours is not always the best investment [11].

Importantly, the context determinants need to be considered as well. Social and cultural support, norms and identity, including those that relate to certain religious, educational or philosophical views can influence attitudes to vaccination [7]. Research has also shown that the social determinants (the circumstances in which people are born, grow up, live, learn and work [12]) affect vaccination uptake, including parental socioeconomic status, number of years in education and ethnicity [13]. Across the WHO European Region there are underserved and marginalized population groups who do not access vaccination services to the same degree as the rest of the population [4, 13, 14]. Context determinants also include how vaccination services are provided, and the way health systems are designed and financed, that can help resolve this inequity [13] and affect vaccination uptake [11].

The WHO recommends health authorities to focus on both individual and context determinants of vaccination behaviours. Indeed, these are complex, multifaceted and operate at multiple levels—intrapersonal (individual determinants), interpersonal, community, institutional and policy (context determinants) [15].

## A comprehensive theoretical model

With this in mind, the approach to ensuring increased uptake must be comprehensive and consider determinants that relate not only to the individual (such as misperceptions, distrust or lack of information), but also to the context (such as social, cultural, institutional and political factors). To avoid missing fundamental challenges, all of the potential barriers (and drivers) to vaccination in the target groups need to be explored. In addition, experience shows that one cannot rely solely on assumptions among health workers or authorities about reasons for low vaccination coverage as these can be challenged or even disproven when barriers are explored in more depth [16]. For example, the influence of anti-vaccination messages on parents can be overrated, whilst the lack of an effective recall/reminder system may be overlooked.

Many health behaviour change models exist which can help health authorities analyse vaccination intentions and behaviours. In the WHO Regional Office for Europe we have adapted the COM‑B model ([17]; the letters stand for Capability, Opportunity, Motivation—Behaviour) for vaccination and are using this adapted model for our Tailoring Immunization Programmes (TIP) approach [18, 19]. The COM‑B model was originally developed for any behaviour (not vaccination per se) by a team of researchers based on 19 frameworks of behaviour change [20].

We chose the COM‑B model because it applies a broad perspective and a comprehensive framework for analysis that includes both individual and context determinants of behaviour. It also provides a logic framework which can be used at all stages: planning research, analysing data, structuring findings and designing theoretically informed interventions for increased vaccination uptake that target the key barriers and drivers identified.

**Fig. 2:**
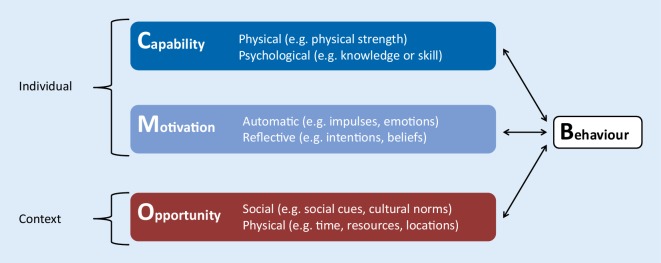
The COM‑B factors with examples for each factor

The core of the model are three factors that need to be in place for any health behaviour to occur: capability, opportunity and motivation. Capability and motivation factors are individual determinants. Opportunity factors are context determinants, that is factors outside of the individual, in the physical and social surroundings. The factors interact; capability and opportunity both influence motivation; and all three factors influence behaviour. Conversely behaviour influences all three factors; in fact, past vaccination behaviours are predictors of future vaccination behaviours [21]. Each of the three factors have two sub-components (see Fig. 2).

## Adaptation of COM-B to vaccination behaviours and TIP

Available evidence related to the determinants of vaccination confirms that all three factors of the model are relevant. Several studies have shown that individual motivation factors such as risk perceptions, confidence, concerns and worry correlate with vaccination behaviours [11]. Individual capability, e.g. in terms of knowledge and information levels, or skills and personal ability to book and follow through on intentions to vaccinate, are equally important barriers or drivers to vaccination [22]. For the opportunity factors, substantial evidence shows that social processes and norms shape vaccination behaviours [11], and that physical factors such as policies, systems, cost and logistics are likewise important determinants for vaccination behaviours [11, 22–24]. However, some adaptation and simplification of the COM‑B model has been made following testing of the model by national immunization programmes in the Region.

Our experience to date has identified that the two dimensions related to motivation (automatic and reflective motivation) and the two dimensions related to capability (psychological and physical) for vaccination are interlinked. For example, thinking about vaccine safety and worrying about vaccine safety would be classified as reflective and automatic motivation, respectively; however, distinguishing between them in the analysis and subsequent design of interventions can be challenging and has not added value. Likewise, the physical skill of a health worker to administer a vaccine is inseparably linked with the psychological knowledge that is required to do this. In an attempt to make the model as simple and easily accessible for all, we therefore decided not to separate capability and motivation in sub-factors.

For opportunity this has proven different. The physical opportunity factor has been shown to be important. Vaccination more than many other health behaviours (e.g. physical exercise, healthy diets, smoking cessation) relies on physical opportunity in the form of a well-functioning public health and vaccination service delivery system as well as appropriate legislation, vaccination supply, qualified staff and sufficient financial resources in the health system. Social opportunity is likewise an important factor for vaccination that is easily distinguishable from physical opportunity, as it relates to social, community and cultural support, values and norms.

**Fig. 3:**
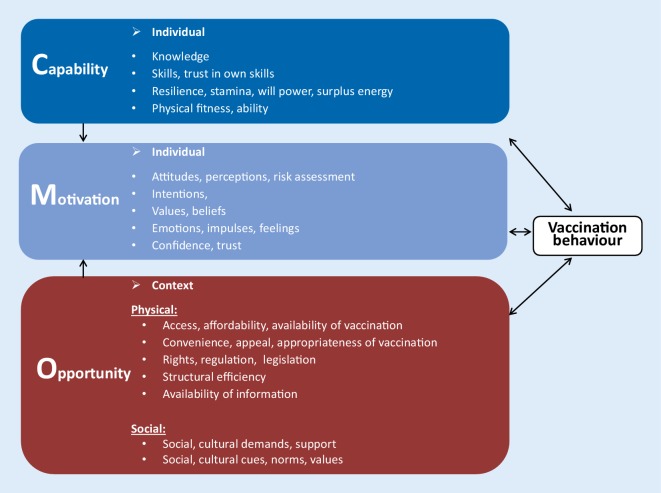
**The** COM‑B model adapted to vaccination by the WHO Regional Office for Europe with examples for each factor. The model has been adapted for use with the WHO Tailoring Immunization Programmes approach

Our vaccination adaptation of the COM‑B model (Fig. 3) therefore:

- Considers capability as one factor directed by both psychological and physical mechanisms
- Considers motivation as one factor directed by both reflective and automatic mechanisms
- Consider opportunity as divided into two factors: physical and social

Evidence shows that the encounter between the vaccine recipient/caregiver and the vaccination provider (doctor, nurse, health worker) is a critical moment in vaccination decision-making [25–27]. Accordingly, we recommend that health authorities explore the barriers and drivers from the perspective of both groups of stakeholders. Infobox 1 shows an example of topics that can be explored for one factor (social opportunity) and two target groups (caregivers and health workers).

### Infobox 1 Example of areas which could be explored related to one factor: social opportunity, for caregivers and health workers

*Caregivers*

- Are they affiliated with a group or community which actively encourages or discourages vaccination (religious, online, philosophical)? How, where, when?
- Do their community leaders (religious, political, social) encourage/discourage vaccination?
- Do health workers promote vaccination and provide the appropriate and needed information and support for vaccination?
- Do their peers actively encourage/discourage vaccination?
- Do they think their peers expect them to vaccinate? What are the social consequences and reactions to vaccination/non-vaccination?
- Is vaccination a social norm and expectation in their community? Is vaccination/non-vaccination accepted by their peers? Do they think their peers vaccinate?
- Is vaccination a topic of debate in their community or among their peers? How, when, where?

*Health workers*

- Are they (generally and for vaccination) supported by their patients and the local community?
- Which demands are they faced with from caregivers and vaccine beneficiaries, and how does this affect them?
- Are they supported by their management where they work; in which way—and do managers actively encourage vaccination?
- Are they supported by their peers; and do peers actively encourage vaccination?
- Are they supported by the local and national government and health authorities?
- Are they affiliated with a group or community which actively encourages or discourages vaccination?
- What are the social consequences and reactions if they do not vaccinate themselves or their patients?
- Who are their role models, and who do they respect and trust when it comes to vaccination?

## Ways to increase acceptance and demand

Informed by the theoretically and evidence-informed insights gained from research with target groups and engagement of key stakeholders (e.g. using the COM‑B model to structure findings), interventions can be designed, implemented and evaluated with the aim to increase vaccination uptake. Some evidence exists related to best practice and proven methods to improve vaccination uptake. However, this is still a fairly new field of science. It is only within the past 10–15 years that vaccine acceptance and demand has developed into a distinct field of research and implementation, and much still needs to be done [28]. The currently available evidence stems from various fields, including psychology, sociology, medical anthropology, social and political science and communication. It is promising, but only few approaches suggested have been sufficiently tested to be deemed best practice. In fact, from the literature on vaccine hesitancy, acceptance and demand, it is not possible to highlight just one strategy or a few specific interventions that, above others, are the most effective to increase vaccination uptake, and interventions with several components seem to be more effective than single-component interventions [29]. A good overview of the available evidence on effective interventions can be found in a few literature reviews related to vaccine acceptance and demand [11, 29–33]. Some key conclusions from these are summarized here.

At the individual level (capability and motivation), some interventions have been shown to increase vaccination uptake, e.g. health workers providing face-to-face clear, balanced information about vaccination risks and benefits ([32, 34, 35]; see Table 1). In addition, many experimental studies have assessed how different types of messages or interventions affect people’s perceptions about vaccination and intentions to vaccinate. However, there is a lack of large-scale implementation of these approaches to successfully show that people’s vaccination perceptions can be changed [11]. Many traditional information and educational tools—such as fact sheets or posters—have been shown to lack effectiveness and have no or little impact on vaccine hesitancy, or even entail a risk to increase hesitancy [29, 36]. Communicating about the risk of diseases may have the power to enhance people’s perceptions of risk, but does not necessarily have the intended effect on intentions to vaccinate [9, 37]. Worse than that, trying to correct misperceptions about vaccination can have the opposite effect, i.e. reinforce the misperception in the person receiving the information [9, 36, 38, 39].

**Table 1:** Examples of interventions that have shown documented impact on vaccination uptake

COM‑B factor	Examples of interventions
*Capability*	Caregivers keeping a copy of their childhood vaccination record at home [41]
	Health workers providing face-to-face clear, balanced information about vaccination risks and benefits and the childhood vaccination schedule [32, 34]
	Health staff training to build skills to support and communicate with caregivers and provide relevant and appropriate information [42]
*Opportunity, physical*	Managers in health facilities trained to provide supportive supervision to staff [43, 44]
	Structured and well-functioning vaccination call and reminder systems [22, 33]
	Minimal direct (e.g. payment for vaccines) and indirect costs (e.g. travel) related to vaccination for caregivers and adults being vaccinated [30]
*Opportunity, social*	Health workers trained in using recommended approaches to address parental concerns and establish an enabling environment for people to make positive vaccination decisions, e.g. applying motivational interviewing techniques and providing a clear provider recommendation for vaccination [25, 42, 45, 46]
	Health workers being an example (e.g. confirm they have vaccinated themselves/their children) to their patients [11, 25]
*Motivation*	Evidence-based decision aid for supporting informed decision-making about vaccination [42]
	Health workers providing face-to-face clear, balanced information about vaccination risks and benefits and the childhood vaccination schedule [33–35]
	Incentives for children/adolescents [30], caregivers [33] or vaccinators, e.g. reimbursement for health-care providers who vaccinate [35]

At the context level (opportunity), there is more documentation for effective strategies for increasing vaccination uptake (examples in Table 1). These include those which facilitate opportunities to vaccinate, through making vaccination easy and convenient, e.g. with an effective recall and reminder system [22, 33], and making vaccination the default behaviour and obvious choice, e.g. by a strong recommendation to vaccinate from the health worker [11], and through ensuring face-to-face interaction between the caregiver and the health worker, which provides reassurance, builds trust and offers the right information [31]. Interventions focusing on social processes as a means to increase vaccine acceptance and demand, such as those related to social norms, moral values or altruism, are a promising field, but also one that requires more evidence to confirm its impact [11, 40].

To illustrate the use of the adapted COM‑B model, some examples of TIP projects from the WHO European Region, their target groups, target COM factors and interventions are presented in Table 2.

**Table 2:** Examples of TIP projects across the WHO European Region

Country	Target groups	Examples of studies conducted	Examples of barriers identified (COM factors)	Examples of interventions recommended based on the evidence
Armenia	Medical specialists who are consulted for advice on vaccination	Interview study with medical specialists	Capability Motivation	Education and training on vaccination Vaccination curricula for medical schools
Bosnia and Herzegovina	Primary care health workers Caregivers	Patient file review study to identify characteristics of sub-optimally vaccinated Interview study with health workers Interview study with caregivers	Opportunity Capability	Implementation of a consistent recall and reminder system across health facilities Strengthened information provision to health facilities Training of health workers
Kyrgyzstan	Internal migrants	Review of behavioural studies Legislation review Focus group evaluation of interventions	Opportunity	Legislation to ensure access to free vaccination Training of and information material to health workers on legislation and rights of migrants
Serbia	Primary care health workers Caregivers	Review of coverage data Interview study with health workers Focus group evaluation of interventions	Capability Motivation	Communication skills training for health workers Information for caregivers
Sweden	Somali community	Review of coverage data Interview study with health workers Interview study with Somali community members	Opportunity: Social Capability	Tailored tools for communication Community meetings Peer support Health worker education
United Kingdom	Charedi Jewish community	Literature review Outbreak and surveillance data analysis Questionnaire survey Interview study with caregivers and key informants	Opportunity	Changes to primary care services to improve access—flexible appointments, proactive reminders, child friendly facilities, reduced waiting times, Charedi nurse working with practices

## Discussion

The growing body of literature and evidence on vaccine acceptance, hesitancy and demand is an important source of knowledge that national immunization programmes can draw on when developing strategies for increased vaccination uptake. It is important to learn from the experience in other countries, and the literature uncovers important successful and less successful practices. However, even if some interventions have been shown to have an impact on vaccination uptake, there is no guarantee they will have an impact in a different context, e.g. where the most important barriers to vaccination are related to a different factor, or where the health system and vaccination service delivery are organized differently. This has three implications.

First, it is recommended that before any intervention is planned, insights into the drivers and barriers to vaccination in the key target groups are gained through empirical data and a situation analysis [29]. These barriers should be analysed to ensure the most effective and cost-effective intervention, targeting the core of the problem.

Second, it is recommended that formative research and subsequent intervention development are both informed by a theoretical model, such as the COM‑B model or other health behaviour change models, as well as a planning framework [29]. Such planning frameworks are available from WHO (TIP [18, 19]) and UNICEF (the Human-Centred Design approach [47]).

Third, the fact that more evidence is needed obliges all stakeholders in the field—researchers and practitioners alike—to contribute to documenting both good and bad experiences. We all have a responsibility to pilot test, monitor, evaluate as well as document and share experiences, anywhere and anytime that any approach to increasing vaccination uptake is being applied.

## Conclusion

In conclusion, overcoming the challenges of achieving and maintaining high and equitable vaccination uptake in all population groups is not a simple or a quick-fix exercise. Fortunately, there is a growing resource of evidence and experience from various settings that we can learn from. However, to truly address challenges in most places requires long-term, diligent and intelligent investment in a multifaceted intervention targeting the core of the problem. To inform such an investment, research with and engagement of the target groups is necessary.
